# Patient-public engagement strategies for health system improvement in sub-Saharan Africa: a systematic scoping review

**DOI:** 10.1186/s12913-021-07085-w

**Published:** 2021-10-05

**Authors:** Samuel Egyakwa Ankomah, Adam Fusheini, Christy Ballard, Emmanuel Kumah, Gagan Gurung, Sarah Derrett

**Affiliations:** 1 Department of Preventive and Social Medicine, University of Otago, PO Box 56, Dunedin, 9054 New Zealand; 2 Center for Health Literacy and Rural Health Promotion, Accra, Ghana; 3 Health Sciences Library, University of Otago, Dunedin, New Zealand; 4 Department of Health Administration and Education, University of Education, Winneba, Ghana; 5 Department of General Practice and Rural Health, University of Otago, Dunedin, New Zealand

**Keywords:** Patient-public engagement, Community engagement, Social accountability, Health system improvement, Health interventions, Sub-Saharan Africa

## Abstract

**Background:**

Actively involving patients and communities in health decisions can improve both peoples’ health and the health system. One key strategy is Patient-Public Engagement (PPE). This scoping review aims to identify and describe PPE research in Sub-Saharan Africa; systematically map research to theories of PPE; and identify knowledge gaps to inform future research and PPE development.

**Methods:**

The review followed guidelines for conducting and reporting scoping reviews. A systematic search of peer-reviewed English language literature published between January 1999 and December 2019 was conducted on Scopus, Medline (Ovid), CINAHL and Embase databases. Independent full text screening by three reviewers followed title and abstract screening. Using a thematic framework synthesis, eligible studies were mapped onto an engagement continuum and health system level matrix to assess the current focus of PPE in Sub-Saharan Africa.

**Results:**

Initially 1948 articles were identified, but 18 from 10 Sub-Saharan African countries were eligible for the final synthesis. Five PPE strategies implemented were: 1) traditional leadership support, 2) community advisory boards, 3) community education and sensitisation, 4) community health volunteers/workers, and 5) embedding PPE within existing community structures. PPE initiatives were located at either the ‘involvement’ or ‘consultation’ stages of the engagement continuum, rather than higher-level engagement. Most PPE studies were at the ‘service design’ level of the health system or were focused on engagement in health research. No identified studies reported investigating PPE at the ‘individual treatment’ or ‘macro policy/strategic’ level.

**Conclusion:**

This review has successfully identified and evaluated key PPE strategies and their focus on improving health systems in Sub-Saharan Africa. PPE in Sub-Saharan Africa was characterised by tokenism rather than participation. PPE implementation activities are currently concentrated at the ‘service design’ or health research levels. Investigation of PPE at all the health system levels is required, including prioritising patient/community preferences for health system improvement.

## Introduction

Despite implementing many health interventions to improve health and health systems in Sub-Saharan Africa, concerns remain about ongoing disappointing health outcomes. A key reason for this may be the inability of health interventions to adequately respond to the perceived needs and interests of the population [1]. A comprehensive review of the extent and nature of community participation and important facilitators and barriers in health systems research for LMICs has not yet focused on Sub-Saharan Africa [2]. Calls have been made for an increased emphasis on engaging communities when implementing health initiatives in Sub-Saharan Africa [3].

The Sub-Saharan Africa region contains less than 10% of the world’s population yet it carries an estimated 24% of the global burden of diseases in both human and financial cost [4]. For instance, 60% of the world’s burden of the HIV/AIDS epidemic is concentrated in Africa with greater majority found in Sub-Saharan Africa [5]. Additionally, other tropical diseases such as malaria, onchocerciasis, schistosomiasis, and lymphatic filariasis continue to adversely affect the region [6]. It is estimated that 90% of the 300–500 million people infected with malaria worldwide each year live in Sub-Saharan Africa [7]. Although improvements have been reported [8], the WHO reports that 19 of 20 countries with the highest maternal mortality rates worldwide are in Sub-Saharan Africa [7].

There is, however, evidence that actively engaging people in planning and implementing major health programmes improves both health outcomes and the health system [9]. Patient-Public Engagement (PPE) has, therefore, been promoted as a key strategy to achieving this. Various PPE reviews conducted in other parts of the world have reported on PPE’s positive effect on health system improvement [10–14]. A review conducted in the United Kingdom (UK) found engagement with lay-volunteers and patients impactful, particularly when designing clinical trials or implementing community-based health programmes [14]. Similarly, another review conducted in Anglo-American contexts and other countries also recommended the need to identify key PPE strategies and situate them within the context of health system levels for effective health policy design [15].

PPE has been defined as the active engagement of citizens, users, carers and their representatives in the development of health care services and as partners in their own health care [16].

Engagement can include: identifying health strategies, setting the health care agenda, planning, selecting the implementation of major health initiatives, and involvement in accountability processes [17–20]. Engagement helps ensure health policies and/or programmes are responsive to the health needs of all groups within the community, particularly, women and children, elderly, adolescents, indigenous and ethnic groups [21]. The 1978 Alma-Ata Declaration on Primary Health Care further states that people have the right and duty to participate individually and collectively in the planning and implementation of health care [22]. Consequently, engagement as a basic human right offers a strategic route to addressing poor health outcomes [9].

Despite the demonstrated advantages of PPE for health, low levels of engagement have been found in Sub-Saharan Africa [23, 24]. Some studies in Sub-Saharan Africa have interventions to be inappropriate for communities, or tokenistic, which may partly account for low levels of engagement [25, 26]. Literature on PPE initiatives in the region does not appear to have been synthesised to provide an understanding of how PPE initiatives have affected health outcomes, health care delivery, or the barriers and facilitators of effective PPE implementation [8, 27–29]. Other reviews of PPE initiatives have found very few PPE strategies have been implemented [30–32]. While these findings are useful, it is also necessary to synthesise and appraise the PPE strategies, particularly, in Sub-Saharan Africa, to describe the health system levels and engagement stages at which PPE is operating and to understand its overall effect on improving the health care system and outcomes.

To address these knowledge gaps, this scoping review aims to identify and synthesise published literature on PPE strategies implemented across Sub-Saharan Africa. Specifically, this paper describes how identified initiatives have impacted on health and health system improvement, and systematically maps identified PPE initiatives onto a framework comprising both health system levels and an engagement continuum.

## Methods

We conducted a systematic scoping review of published peer-reviewed research articles published between 1999 and 2019 reporting PPE strategies aimed at improving health systems in Sub-Saharan Africa. The detailed scoping review protocol has been published [33]. Briefly, we followed Arksey and O’Malley’s guidelines for conducting this scoping review [34–36]. We also reported results using the Preferred Reporting Items for Systematic Reviews and Meta-Analysis extension for scoping reviews (PRISMA-ScR) [37]. The scoping review questions, informed by the Population, Concept and Context (PCC) framework [37] were:

1. “What PPE strategies for improving health systems have been implemented in Sub-Saharan Africa and reported in peer-reviewed literature?
2. What are the outcomes of these identified PPE strategies and strategies for health system improvement in Sub-Saharan Africa?
3. What are the current knowledge gaps about PPE in Sub-Saharan Africa?” [33] (p.4).

### Data analysis

We employed thematic framework analysis to analyse the literature identified [38–40]. All studies were mapped onto an engagement continuum framework (Table 2; SEA, AF and SD) adapted from Bombard et.al, Gurung et.al, Ocloo and Matthews, Bate and Robert [12, 54–56] and applied in this study to understand whether engagement strategies have been implemented at the ‘consultation’, ‘involvement’ or ‘partnership and shared leadership’ stages. The settings in which these PPE strategies were implemented were also identified and mapped onto the framework. The mapping of the studies onto the framework was independently verified by two members of the research team (SD and AF).

**Table 2:** Mapping identified papers to the Engagement Continuum and Service Levels Framework

Engagement Context	Type of Engagement		
	Consultation	Involvement	Partnership and Shared Leadership
Research projects	[1, 41] (*n* = 3)	[29, 42–44] (*n* = 3)	(*n* = 0)
Individual treatment	(*n* = 0)	(*n* = 0)	(*n* = 0)
Service design	[3, 45] (*n* = 2)	[28, 31, 46–53] (*n* = 10)	(*n* = 0)
Macro policy/ Strategic	(*n* = 0)	(*n* = 0)	(*n* = 0)

## Results

**Fig. 1:**
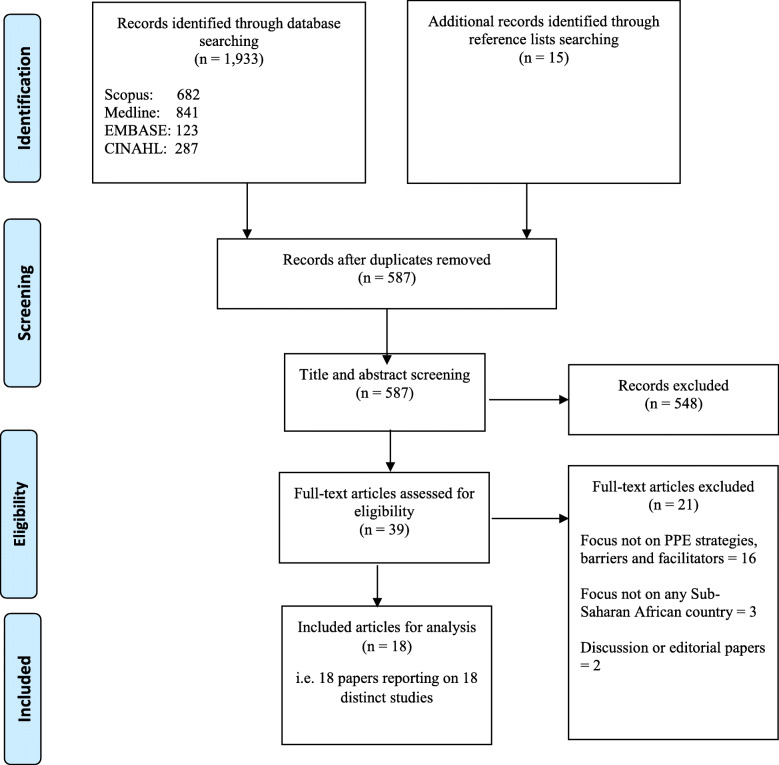
PRISMA-ScR flow chart summarising literature search and selection of articles [37]

A total of 1948 articles were initially identified; 1933 through database searching, and 15 through hand-searching of the reference lists of identified articles (Fig. 1). Following removal of duplicate records, the number reduced to 587. A further 548 papers were excluded following the title/abstract screening, leaving 39 papers for full text screening. After applying the inclusion and exclusion criteria, 21 articles were excluded (16 were not focused on PPE strategies, barriers or facilitators; 3 were not focused on sub-Saharan Africa; and 2 were discussion or editorial papers). Therefore, 18 articles were retained for final synthesis.

### Characteristics of the included studies

The 18 eligible papers reported on 18 discrete studies undertaken in 10 Sub-Saharan African countries (Table 1). Most studies (*n* = 14) used qualitative methods [1, 3, 28, 29, 31, 41–43, 46–51], one was quantitative [45], and three used mixed methods [44, 52, 53].

**Table 1:** Summary of publications on PPE strategies in Health System Improvement in SSA

Author (s) and Year of publication	Country of origin	Aims/Purpose	Study Design and Sample	Intervention setting	Engagement Strategies	Outcome (s)	Main finding (s)
Adongo et.al. (2013) [46]	Ghana	To assess the impact of male involvement in Family Planning in Northern Ghana	Qualitative descriptive design. 90 participants via 12 focus groups discussions 59 In-depth interviews	Maternal Health Services (Family Planning)	Focus groups, Community educational Workshops	Spousal approval for women in the use of contraceptives; CHPS effect on male involvement in Family Planning.	Males were much involved in family planning activities in communities with functioning CHPS compared to those without functioning CHPS. Spousal approval was still relevant for women’s use of contraceptives.
Angwenyi et.al., (2014) [44]	Kenya	To share community engagement experiences in an on-ongoing paediatric malaria vaccine trial conducted in three study sites of Kilifi County, Kenya.	Mixed method descriptive design 25 participants via focus groups 32 participants via in-depth interviews 200 through observational surveys	Malaria Vaccine Trial	Interviews, Focus groups, Observation of Community Engagement activities	Daily engagement with community stakeholders and the Trial teams’ goals for community engagement before Malaria Vaccine trial	Compared with the trial teams’ community engagement goals, regular engagement with community stakeholders had different expectations and goals. Engagement with community stakeholders was effective in reducing misconceptions about the vaccine trial, thereby contributing positively to a successful trial of the vaccine.
Baatiema et al., (2013) [28]	Ghana	To explore the PPE process of a community-based health planning and services programme in the Upper West Region of Ghana and evaluate the perspectives of the local stakeholders on their participation in the programme and its impact on health care delivery.	Qualitative Study applying Spider-gram theory to measure extent of participation 17 participants via in-depth interviews 17 participants involved in 2 focus group interviews	Primary Health Care	Interviews, Focus groups Community Meetings	Use of community resources; CHPS integration with pre-existing community structures; aligning CHPS with community interest.	Engagement with community become sustained and more effective by acknowledging and using community resources and integrating CHPS with pre-existing community structures as well as aligning health interventions with community interest.
Campbell et al., (2008) [3]	South Africa	To report on the perceptions of the community on a 3-year programme that seeks to promote grassroots’ responses to HIV/AIDS – mainly through rural health volunteers in KwaZulu-Natal, South Africa.	Qualitative study involving 12 participants via in-depth interviews and 34 participants involved in 5 focus group discussions	HIV/AIDS Prevention	Interviews Focus Groups Meeting with community leaders	Increased grassroot support for volunteers in HIV/AIDS home nursing care.	The project significantly enhanced community confidence in health volunteers and also contributed to increased community knowledge and acceptance for home nursing care for people living with HIV/AIDS.
Chilaka, (2005) [45]	Burkina Faso, Ghana, Nigeria, Tanzania & Uganda	To use quantitative values to measure and compare levels of community participation in the Roll Back Malaria programme in five Sub-Saharan African countries	Quantitative cross-sectional analysis of database using Spider-gram theory to assess 503 reported malaria cases across the studied countries (excluding Burkina Faso)	Malaria Control	Secondary data Analysis of Roll Back Malaria Programme	Community participation present at all levels of the RBM programme, Varying degree of community participation existed in the RBM programme.	Results highlighted that higher degrees of participation, among other factors resulted in improved incidence of malaria under the Roll Back Malaria programme
Dougherty et al., (2018) [52]	Ghana	To examine how a Community Benefit Health (CBH) programme influenced the outcomes of maternal health services through continuous sustenance of community-level support among the social networks of women.	Mixed method study with 1746 participants involved in questionnaire survey and 183 participants via in-depth interviews & focus group discussions	Maternal Health Services	In-depth interviews, Focus groups, Educational meetings with community leaders	Maternal health behavioural response to CBH interventions; Male engagement in maternal health.	Results showed improved maternal health outcomes such as antenatal/postpartum care, birth attendance and breastfeeding following enhanced engagement to change community and spousal attitudes towards maternal health issues.
Gregson et al., (2013) [1]	Zimbabwe	To investigate if PPE or Community grassroots participation resulted in increasing HIV Testing and Counselling services in Zimbabwe	Prospective cohort study involving 5260 participants interviewed in 2 consecutive rounds of cohort survey	HIV/AIDS Prevention	Interviews Educational programmes	Uptake of HIV Testing and Counselling, partnership with organisations for community support	Results showed increased HIV testing and Counselling uptake services for community organisations due to grassroot participation compared with non-community organisations
Kamanda et al., (2013) [47]	Kenya	To describe the approaches and principles of Community-Based Participatory Research through harnessing grassroots power in conducting public health research in Sub-Saharan Africa.	Randomised Controlled Trial with semi-annual assessment of 3130 participants	Community Public Health Research	Community Advisory Boards (CAB) Household interviews	Adaption of Community-Based Participatory Research in implementation of community health programmes and public health research Cultural and community relevance in shaping public health research and interventions	Community engagement effectively shapes public health research design and increases community participation in subsequent implementation of community-based health interventions
Mafuta et al., (2015) [48]	Congo	To explore how health care providers respond to concerns of women through an existing social accountability mechanism in a local setting.	Exploratory study involving two health zones with 48 participants interviewed	Maternal Health Services	Interviews, forming Health Committees and CABs	Varied perception of health providers’ responsiveness, lack of support for women’s participation in maternal health despite existence of many local community health-related groups	Results showed that, most women did not have voice to participate in maternal health issues. Among the factors found include; absence of procedures to express views, lack of knowledge, fear of reprisal, ethnicity, power and status.
Musesengwa and Chimbari, (2017) [29]	South Africa & Zimbabwe	To document the experiences of members in Community Engagement processes during a project implementation in two countries	Qualitative case study approach involving 102 participants via focus groups and 66 participants via interviews	Malaria and Bilharzia	Geospatial disease and vector Mapping, Focus group, Participatory Rural appraisal (PRAs) workshops, Biomedical techniques, CAB	Community experience in community engagement process, Research naïve community in public health research and implementation of health programmes.	Results showed that continuously soliciting views and preferences from main stakeholders significantly contributes to the engagement process. Also, compared with research experienced communities, research naive communities can significantly contribute to research and community engagement process
Ntshanga et al., (2010) [31]	South Africa	To strengthen community mobilization, awareness, education and involvement to improve TB control by building community-health sector partnership through the establishment of Community Advisory Board.	Cross sectional study with a total of 140 participants involved in 2 Consultative workshop	Tuberculosis Control and Research	Stakeholder workshops through CAB	Mechanisms for community consultation and participation	Results revealed low incidence of TB was due to the regular community involvement in TB control activities. CABs were found to be effective in facilitating community involvement in patient care.
Person et al., (2016) [49]	Tanzania	To describe, using Human-Centred Design in Community co-designed process to prevent and control Schistosomiasis. It also aimed to explore how local knowledge, creativity and experiences could be used to design community-owned structural and behavioural interventions to reduce the spread of Schistosomiasis.	Cross sectional study involving 5 focus group discussions with community figureheads, 35 school-based discussions with children, 25 interviews with teachers and 16 parents	Schistosomiasis	School-based education and training, focus groups, interviews	Sustainable PPE strategies for controlling Schistosomiasis; implementing PPE using Human-Centred design	The outcome of the study revealed that community co-designed process with emphasis on Human-Centred Design principles of PPE ensures a more sustainable and effective interventions for controlling Schistosomiasis.
Riehman et al., (2013) [41]	Kenya	To examine the impact of Community-Based Organisations on community and individual level health outcomes; focusing on perceptions, awareness, knowledge, sexual risk behaviours of HIV/AIDS.	Quasi-experimental cluster design with multi-method data collection involving 4378 adult respondents	HIV/AIDS prevention	Advocacy workshops, Community meetings, educational workshops	Higher levels of Community-Based Engagements, PPE strategies for Community-Based Organisations including sexual risk behaviours, awareness programmes, and social transformation (gender ideology and social capital).	Study revealed communities with more Community-Based Organisations engage more and therefore tend to have less incidence of HIV/AIDS compared with those with less Community-Based Organisations.
Sakeah et al., (2014) [50]	Ghana	To examine the role played by community leaders and residents during the implementation of skilled delivery programme and its effect on improving maternal health care in a Ghanaian community.	Intrinsic case study design with a qualitative methodology involving 29 health professional and community stakeholders interviewed	Maternal Health Services	Homes visits through CHV, CHW and interviews, Traditional authority involvement	Community members role in promoting skilled delivery in CHPS zones; mutual collaboration and engagement between health professionals and community members	Study revealed community members are key to promoting skilled delivery and reducing maternal mortalities and pregnancy related complications. Relationship between community Health Volunteers and Traditional Birth Attendants were found to be key in providing health education on skilled and safe delivery.
Tancred et al., (2017) [53]	Tanzania	To examine the complexity of community-level quality improvement in health by building capacities of community members to use quality improvement behavioural change towards enhancing maternal and newborn health in Tanzania.	Mixed method involving 83 participants interviewed or involved in focus groups, and quantitative data from secondary sources	Maternal and Newborn Health Services	Educational meetings with Health Volunteers, focus groups & interviews	Performance implementation scores to rank communities; Changing health seeking behaviours and uptake of community level maternal and newborn health services, regular education around quality improvement in maternal health care.	The study results revealed facilitators of PPE as ones which were most prevalent in high-performing communities, whilst the barriers were those which were lacking in these high-performing communities. The identified facilitators and barriers are key to influencing behavioural change to improve maternal and newborn health
Tindana et al., (2011) [43]	Ghana	To describe the community engagement practices frequently used during implementation of health projects or research through the Navrongo Health Research Centre of Ghana, and to identify the underlying cultural norms that informed those community entry practices	Qualitative case study design involving 116 participants in focus groups and 20 involved in in-depth interview	Public Health Research	Interviews, focus groups and meetings with traditional rulers	Social mappings; Traditional community engagement mechanisms; Community confidence in health professionals, and gender inequities.	The study found that using existing traditional structures in a community reduces social disruptions during implementation of community-based health interventions.
Yeboah and Jagri, (2016) [51]	Ghana	To identify the factors that constrain or facilitate community engagement activities during the implementation of the community-based Health Planning and Services (CHPS) programme in the central region of Ghana.	Qualitative case study design involving 103 participants via questionnaire survey, 8 participants via interviews, 1participant via informal discussion.	Primary Health Services/Maternal Health	Health committees, interviews, Community health durbar	Community support during post implementation phase in a community-based health intervention; Assessing local community’s commitment in participation on spider-gram theory; Establishing framework to define community role, expectations and responsibility prior to implementing community-based health interventions.	The study found that having clearly defined shared leadership and partnership role between health authorities and communities prior to implementing community-based health intervention is key to reducing post-implementation tensions and conflict that can disrupt achieving the core goals of the programme.
Meiring et al., (2019) [42]	Malawi	To describe community and stakeholder engagement practices prior and during a typhoid conjugate vaccine trial; drawing lessons from the challenges and its impact on the health outcomes.	Qualitative research design within a Randomised Controlled Trial with 380 participants involved in Focus groups, interviews, other engagement meetings	Typhoid Vaccine Trial	Focus Group Discussions, School-based meetings, Community Advisory Group meeting and Media engagements	Involving wide range of stakeholders; starting community engagement early and throughout implementation phase; adequate allocation of resources to support community engagement; use of broad range of complimentary engagement activities.	The study results found there was improved awareness and high turnout for the vaccine trial following an enhanced engagement with local government and community leadership as well as employing multiple channels of communication.

### Study findings

The following major categories of PPE strategies implemented across Sub-Saharan Africa were identified: (1) traditional leadership support and collaboration; (2) community advisory boards; (3) community education and sensitisation; (4) community health volunteers/workers; and (5) aligning and embedding PPE within existing community structures. Additionally, this review mapped the identified studies onto an engagement continuum framework to understand the current focus of PPE implementation in Sub-Saharan Africa, including how strategies have been applied to health system improvement.

### Traditional leadership support and collaboration

Eight studies from six sub-Saharan African countries found traditional leadership support and collaboration was critical to effective PPE [28, 29, 42–44, 47, 50, 51]. However, the studies reported different forms and timings of traditional leadership support and engagement.

For instance, a study conducted in Ghana, reported that seeking early pathways to PPE by making decisions with traditional leaders, served to reassure community members that their cultural values were respected [43]. It also ensured engagement strategies were culturally sensitive as it reduced other social interruptions, which may affect the introduction of health interventions in the community [43, 47].

Studies found the use of traditional channels of communication (e.g. local dance, drama, and iconography) enhanced community engagement [29, 50, 51]. Additionally, using existing local traditional structures to implement health interventions [42, 44], and employing traditional systems (e.g. sub-chieftaincy structures) for post-implementation sustenance of health intervention programmes [47, 50, 51], were central to effective implementation.

### Community advisory boards

Community Advisory Boards (CABs; sometimes referred to as Community Health Committees, Community Advisory Groups, Community Workers Groups or Community Stakeholder Partnerships) were another strategy used to promote community health and develop people-centred health care systems across several Sub-Saharan African countries [28, 29, 31, 42]. CABs are informal advisory groups that seek community opinions or views from multiple stakeholders through focus groups, community meetings, interviews or suggestion boxes to promote people-centred health care services [29]. CABs usually included a range of representatives from the community and/or other health sector stakeholders [31]. In this review, seven articles reported findings from 10 Sub-Saharan African countries related to CAB [28, 29, 31, 42, 44, 48, 51]. These included comprehensive processes for establishing CABs such as ensuring adequate representation from all minority groups in the community [31]. It was also found to be important to avoid underrepresentation or misrepresentation in CABs, by using transparent selection processes to avoid monopolisation of CABs by influential individuals [28, 51]. Training and empowering CAB members to understand the health care system as well as the PPE programme were also important [28, 31]. Communication training was also critical to ensure programme objectives could be communicated to the community, and that feedback from the community was heard [28, 31].

Significantly, CABs helped develop trust and acceptance from local communities and offered an opportunity for communities to directly comment, critique and assist health professionals in developing people-centred health care services [31].

CABs as a PPE strategy have a number of advantages. They were found to be highly effective in urban towns where traditional leadership structures had less influence than in the villages and peri-urban areas [42]; served as a source for representing the voice of the community [31]; helped ensure the aims of health programmes meet local needs of the community [42]; and communicated relevant health system information to the community [29]. Other benefits of CABs included monitoring health programme progress and providing regular updates [44, 48, 51]; promoting active community participation in new health interventions [42]; and ensuring the implementation of new community-based health interventions were sensitive to the values and practices of the community [31, 42].

A South African study found continuation of CABs, beyond the duration of a PPE implementation, was important [31]. For instance, as noted from the PPE experience in a typhoid conjugate vaccine trial in Malawi, among the key successes of the programme was its ability to empower and sustain the activities of the CAB beyond the vaccine trial phase, to continue providing regular updates from the community including reports on adverse drug reactions from the vaccine trial [42]. It was, therefore, recommended to avoid using CABs as an ad hoc PPE strategy for implementing community-based health programmes, but rather to sustain CABs to maintain links between the community and health care providers [31].

### Community education and sensitisation

Evidence of using community education and sensitisation for PPE was reported in all 18 eligible studies - although different approaches were used. These include formal approaches such as health care institutions forming partnerships with community-based organisations (CBOs) [41]; community meetings, workshops and public durbars [1, 3, 28, 42, 44, 53]; training advertisements on radio, television and newspapers [31, 42, 52]; and other informal approaches such as presentations through drama [29]; wearing programme regalia (such as shirts, cups, flyers among others) [29, 42, 43]; and organising community sports and social events to promote health programmes [29, 43].

CBOs such as religious bodies, non-governmental organisations (NGOs) and other key community-based government agencies such as district and municipal assemblies [29, 31, 41], helped reduce the late presentation of cases to health facilities often attributed to spiritual causes [41]. Engagements with other key governmental organisations such as local assemblies and NGOs were also recommended to provide stronger collaboration to effectively engage communities, as well as influencing PPE policy direction, particularly, at the governmental level [31, 41].

The scoping review found community education and sensitisation programmes in Sub-Saharan Africa to have focused mainly on CABs or community representatives [28, 48]; health volunteers/workers [3, 44, 51]; traditional rulers [43]; CBOs [41]; and health professionals [47, 48]. Community liaison officers have also been identified as an important group to include in the education and sensitisation programmes [29].

Community education strategies were noted to be effective particularly when targeted at specific audiences, or were directed towards achieving particular aims and objectives of a health programme [52]. Therefore, this PPE strategy was suggested to be aligned with the cultural practices maximising community cooperation [43].

### Community health volunteers

Community health volunteers (CHVs), also known as community health workers have long been recognised to play a significant role in community participation. CHVs are lay health workers who voluntarily support the delivery of health care services at the community level by providing non-specialist basic health care services to communities, without receiving regular salary or a confirmed position within the health system [57]. The scope of their work usually includes health promotion, vaccination, bed-net distribution, prenatal care and basic non-clinical health support for people with chronic health diseases such as HIV/AIDS and tuberculosis [57–59].

CHVs have been involved in implementing community-based health programmes across many Sub-Saharan African countries [60]. Fourteen of the eighteen articles [3, 28, 29, 31, 41, 44, 46–48, 50–53] reported on CHVs role in PPE. CHVs have been found to be particularly important for rural and socioeconomically deprived communities in Sub-Saharan Africa [3, 46, 53].

CHVs have been used to improve the early diagnosis and treatment of malaria, diarrhoea, pneumonia among others by ensuring affected community members are referred promptly to health facilities for early treatment [3].

CHVs were found to easily gain the trust of the public and patients due to their close working relationships with communities [28, 41, 44]. Studies also reported that CHVs help ensure PPE information resonates with the cultural practices and health care services of the community [3, 31, 46].

CHVs were found to be effective for both cost reduction and reducing community-related barriers to PPE initiatives [51, 53]. Therefore, CHVs have been recommended as key PPE approach to augment the shortage of professional health workers in Sub-Saharan Africa [3, 29, 52].

In addition, a number of factors were identified to influence performance of CHVs. These include a range of contextual factors such as geographical access challenges [3, 28]; socio-cultural norms [47, 52]; financial constraints [51]; and functionality of the health system policy [47, 52]. Thus, Dougherty et al. recommended the use of CHVs as a key component of any PPE approach that aims to change community behaviour (e.g. reducing resistance to the use of contraceptives) [52].

Various factors help account for the widely reported impact of CHVs in Sub-Saharan Africa, including the community-focused process of selecting CHVs [47], quality training given to CHVs [3], and adequate supervision [3, 50].

### Aligning and embedding PPE within pre-existing community structures

Aligning PPE to pre-existing community structures has been found to be important for building community trust, as well as avoiding the duplication of PPE structures such as CABs or CHVs groups that may inadvertently end up competing with each other rather than working together towards a common goal [44]. Of the many PPE strategies, working within community structures has been used extensively in Sub-Saharan Africa [3, 28, 31, 43, 44, 47, 48, 51]. For instance, a randomised control of paediatric malaria vaccine trial in Kilifi, Kenya, integrated all pre-existing PPE decision making structures, including local assemblies, traditional authorities, CBOs and CHVs [44]. Another study indicated how working with pre-existing community structures for PPE was time-saving and cost effective compared to establishing new or parallel PPE structures [47].

In a study conducted in the Upper West region of Ghana, the success of a community-based health planning and services (CHPS) programme was largely attributed to the programme’s ability to absorb all pre-existing community structures such as the community unit committees, CHVs and traditional birth attendants. This to a large extent averted a possible confrontation or conflict between these existing community structures and the CHPS programme [28]. Other studies have also found this approach successful [43, 48, 51]. Although this strategy has been criticised for difficulties of implementation [31], it has been recommended as an effective PPE strategy during the introduction of new interventions such as vaccine trials or health programmes; particularly when programmes may result in community resistance or stigma (e.g. HIV/AIDS programmes) [3].

### Continuum and levels of patient-public engagement

In addition to describing how PPE initiatives affected health and health system improvement in Sub-Saharan Africa, this paper also aimed to map PPE initiatives onto a framework comprising both health system levels and an engagement continuum. This provides an understanding of the current focus of PPE in Sub-Saharan Africa, particularly by situating the identified PPE strategies within the context of health system improvement as recommended by others [15].

The continuum was adapted from Arnstein’s ladder of citizen participation which contained eight rungs of engagement stages. In our paper, we have included three main stages: consultation, involvement and partnership/shared leadership as previously applied by other scholars in different settings [12, 54–56]. The first stage on the continuum, ‘consultation’ has been described [12] as the weakest form of engagement providing patients/public with an opportunity to express their opinions/views, or as a means of disseminating information about the health care system. The ‘involvement’ stage, which is next on the engagement continuum, allows patients/public to express their views and become involved in decision making process while not being part of the final decision-making process. The last stage on the continuum is ‘partnership and shared leadership’ which is generally characterised by shared power and responsibility among health professionals and public/patients working together as partners to design, manage and improve health care services [12].

Most studies identified in our scoping review (*n* = 13), were categorised as ‘involvement’ activities, whilst the remaining (*n* = 5) were categorised as ‘consultation’ (Table 2). None of the studies were categorised as ‘partnership and shared leadership’ activities. For instance, in the consultation category, Gregson et.al conducted a study in Zimbabwe to investigate if PPE resulted in increasing HIV testing [1]. This PPE approach was categorised as ‘consultation’ because members of the community were mainly educated about the importance of HIV testing. They were neither asked about their experiences nor what was important to them as major stakeholders in improving HIV testing [1]. Similarly, another study conducted in South Africa that investigated grassroot perceptions about interventions to support health volunteers was also categorised as ‘consultation’. This was because the community ended up receiving information on how to support the health volunteer programme without any assurance of their experiences, concerns and suggestions being considered to improve the design and implementation of the health volunteer programme [3]. In Kilifi, Kenya, a study investigated community experiences during a malaria vaccine trial. There, the engagement moved from the ‘involvement’ category to ‘consultation’ over the course of the study [44]. This was because although the trial included feedback processes during the initial stages, this decreased overtime and the researchers reported that engagement had not worked as well as intended. The community ended up mainly receiving information intended to clarify misconceptions about the perceived negative effects of the vaccine, without necessarily being able to share their experiences, concerns or suggestions, or having their feedback contribute to improving the design of this community-based vaccine trial [44].

Overall, 13 studies were categorised as ‘involvement’. Most involved feedback from the community or individuals to improve PPE strategies, but did not involve the public/patients in the final decision-making process. For instance, in a study assessing the impact of male involvement in family planning services in Northern Ghana, it was revealed that feedback and suggestions received from the community were mainly used to improve the PPE design [46].

However, the decision to respond (or not) to feedback was not in partnership with the community but was decided by the health care authorities. From the identified studies, none was categorised within the ‘partnership and shared leadership’ stage of the continuum.

Additionally, all identified studies were categorised according to the levels of the health system activity where the PPE initiatives were primarily located [61]. The three-tier categorisation comprised the individual person/patient treatment (e.g. individual patient attendance at health facilities, online patient portals or direct care of individual patients), service design (e.g improving overall service design or changing how the service responds to health interventions) and macro policy/strategic levels (e.g. national government PPE policies and strategies for improving overall health system). A fourth category included PPE activities occurring in the context of ‘research projects’ (e.g. PPE within community-based health research programmes or randomised clinical trials). All included studies (*n* = 18) investigated PPE either in the context of PPE within research projects (*n* = 6), or PPE at the ‘service design’ level (*n* = 12). None investigated engagement at the ‘individual treatment’ or ‘macro policy/strategic’ levels (Table 2).

## Discussion

To the best of our knowledge, this scoping review is the first to systematically identify various PPE strategies implemented across Sub-Saharan Africa and map onto a framework of engagement to assess the current focus of PPE in Sub-Saharan Africa. Overall, our review identified five main PPE strategies implemented across Sub-Saharan Africa: (1) traditional leadership support and collaboration, (2) formation of CABs, (3) community education and sensitisation, (4) CHVs and (5) aligning and embedding PPE within pre-existing community structures. These PPE strategies were demonstrated in 10 Sub-Saharan African countries: Burkina Faso, Congo, Ghana, Kenya, Malawi, Nigeria, South Africa, Tanzania, Uganda and Zimbabwe.

Although a few previous reviews have reported on PPE strategies, these did not focus on PPE’s role in health system improvement [26, 31, 54]. This review has examined PPE activities in the context of improving health systems. We found that traditional leadership support and CABs were the most widely used and effective PPE strategies across Sub-Saharan Africa. As found in previous studies, traditional leaders play important role in community development and are highly revered and regarded in Sub-Saharan Africa as custodians of traditions, values, culture, laws, religion and leaders also serve as reminders of pre-colonial sovereignty [43, 62–64]. Therefore, in most village communities, for instance, the traditional leadership system is more readily acknowledged by local people than formal governmental structures [65]. Thus, the findings of this review, as in past studies [66, 67] support seeking community entry approaches through traditional leaders to ensure PPE activities are endorsed, before communicating these activities to the wider community [43, 68–71].

While important, this strategy appears more in village and peri-urban communities rather than in urban cities where traditional leadership has a more limited role [42, 43]. In urban areas, CABs have been widely used as an effective alternative to traditional leadership for PPE. Advantages of CABs include helping to ensure PPE programmes are sensitive to the cultural needs of the people; meeting local needs; and ensuring proper communication of information to avoid misrepresentation.

Although traditional leadership and CAB strategies have been widely used in Sub-Saharan Africa [72, 73], the present review also identified some key weaknesses. First, working through the traditional leadership structures can be expensive and time-consuming which can affect the timely implementation of PPE [43]. For instance, the local customary processes of visiting a chief and the cost of buying customary gifts, may slow PPE implementation. However, compared to the overall costs of PPE programme implementation, investing these time and costs early on in a new PPE activity are recommended. Further, CABs may not successfully reach all sectors of the community. For example, there may be cultural and language barriers among CAB members and/or low levels of awareness of CABs in the communities [28, 42, 44].

In contrast with past reviews [2, 12, 15], our review focused on how identified PPE strategies were effectively implemented in different Sub-Saharan African health systems and communities. For instance, a systematic review of patient engagement in health priority setting identified CABs as an important PPE strategy without giving further details on how and when this strategy can be most effective [74]. All studies identified in our review were mapped onto a framework of engagement and health system levels to assess the current focus of PPE in Sub-Saharan Africa. The mapping also provides understanding of the levels and stages at which these PPE strategies have been implemented for health system improvement. Overall, most studies described engagement activities categorised as ‘involvement’; with a few studies reporting PPE engagement categorised as ‘consultation’. No PPE activities were categorised as ‘partnership and shared leadership’. This accords with past reviews identifying PPE activities for health system improvement as ‘tokenistic’ rather ‘genuine’ participation [12, 55]. Although other studies have argued against necessarily always aiming for the higher levels of engagement [12, 54], there is a need to try to move away from tokenistic approaches towards prioritising patient/community preferences, and moving to co-design for significant health service improvement [12, 13].

Additionally, PPE studies in Sub-Saharan Africa were all identified at either the ‘service design’ level of the health system or were focused on engagement with specific health research projects. None of the identified studies investigated PPE at the ‘individual treatment’ level or ‘macro policy/strategic’ level. This finding is, however, in contrast with past reviews that have mostly reported PPE at the ‘individual treatment’ level rather than the ‘service design level’ [12, 54]. For instance, an international systematic review of patient engagement to improve health care quality found a large number of PPE studies at the ‘individual treatment’ level with only few located at the ‘service design’ level [54]. None of the identified 18 studies in our review had focused on PPE at all levels of the health system. For instance, in a study conducted in Malawi, a PPE programme directed towards improving community acceptance for a Typhoid Vaccine Trial, did not also consider individual patients’ experiences with the health programme [42]. Similarly, a Ghanaian study investigated health system engagement with communities during the implementation of a CHPS programme without also focusing on individual patients’ experiences [28]. However, as noted in previous studies, investigating PPE at all levels of the health system may provide a more effective understanding of PPE’s overall effect on health system improvement [56, 75].

### Identification of gaps and recommendation for future research

One key aim of scoping reviews is to identify gaps in knowledge which require further research [35]. Our review has identified several knowledge gaps. First, it was identified that most PPE studies in Sub-Saharan Africa have mainly focused on PPE in the areas of ‘service design’ or health research; mostly in advance of implementing new health interventions. While this is important, it is also crucial to have strong PPE activities across all levels of the health system including ‘individual treatment’ and ‘macro policy/strategic’ levels to provide a strong framework for implementation of health programmes and improvement [56, 75]. Further primary research investigating PPE at all key levels of the health system is warranted.

In addition, our review findings also indicate the importance of future PPE initiatives in Sub-Saharan Africa utilising strategies found to work well; particularly, traditional leadership support and CABs.

### Limitations of the study

Our review focused on only peer-reviewed articles; meaning some PPE studies reported in the grey literature will not have been included. Additionally, searching non-health sciences databases may have identified other relevant published articles. Also, despite the wide range of search terms used in different databases, this review may have missed some terms relevant to PPE in Sub-Saharan Africa. Because this review included only published English language articles, it may have missed some Sub-Saharan African PPE articles published in other languages. Lastly, our review focused on initiatives used when implementing PPE activities, the degree of engagement and levels of the health system. Further research is also required to understand the various barriers and facilitators of PPE in Sub-Saharan Africa. Further analysis of the identified papers in this scoping review is now underway to address this gap.

Despite the acknowledged limitations, a strength of this review is the focus on findings of direct relevance and importance to Sub-Saharan African countries and health systems. Further, the strategy used in this scoping review was comprehensive in reviewing public health and health sciences databases to identify PPE strategies for health system improvement in Sub-Saharan Africa.

## Conclusion

Our review found that traditional leadership support and collaboration, formation of CABs, community education and sensitisation, CHVs, and aligning and embedding PPE within pre-existing community structures were the main PPE strategies employed in Sub-Saharan Africa for health system improvement. The review also found traditional leadership support to be an effective and widely used PPE strategy in most village communities, whereas CABs were found to be effective in peri-urban and urban communities. We recommend future PPE initiatives in Sub-Saharan Africa consider these strategies – and dedicate resources to fostering these important strategies in the early phases of PPE activity planning, design and implementation.

Again, although previous studies have reported some PPE strategies, these have not focused on PPE’s role in health system improvement. Our review has identified and examined a key number of PPE strategies and their role in health system improvement, including analysing the health system levels and engagement continuum stages at which these strategies are functioning. PPE studies in Sub-Saharan Africa were mostly identified at the levels of ‘service design’ or health research. We therefore recommend future PPE studies focus on engagement across the range of health system levels to provide knowledge about the development of strong frameworks facilitating easy implementation of community-based health programmes throughout the sector.

Finally, for PPE to achieve enhanced opportunities for improving health systems, there is a need to move from more tokenistic approaches towards genuine participation. Hence, there is a need to prioritise patient and community preferences in the design and implementation of health interventions or programmes to achieve significant health system improvement in Sub-Saharan Africa.

## Data Availability

All reviewed papers analysed during this study are included in this manuscript and the selected peer-reviewed studies are summarised in Table 1.

## Abbreviations

PPE: Patient-Public Engagement
LMICs: Low- and middle-income countries
CABs: Community Advisory Boards
CBOs: Community-based organisations
NGOs: Non-governmental organisations
CHVs: Community health volunteers
CHPS: Community-based health planning and services
